# Safety Doesn’t Happen by Accident: A Longitudinal Investigation on the Antecedents of Safety Behavior

**DOI:** 10.3390/ijerph17124332

**Published:** 2020-06-17

**Authors:** Greta Mazzetti, Emanuela Valente, Dina Guglielmi, Michela Vignoli

**Affiliations:** 1 Department of Education Studies, University of Bologna, 40126 Bologna, Italy; emanuela.valente2@unibo.it (E.V.); dina.guglielmi@unibo.it (D.G.); 2 Department of Psychology and Cognitive Science, University of Trento, 38068 Rovereto, Italy; michela.vignoli@unitn.it

**Keywords:** safety behavior, safety climate, risk perception, construction worker, safety knowledge

## Abstract

Research recognizes the shared perceptions of the priority attributed to safety in comparison to other organizational goals (i.e., safety climate) as a potential antecedent of safety behavior among construction workers. This type of climate can dismantle barriers to the promotion of effective strategies to mitigate workplace hazards. On the other hand, the current understanding of the underlying process that links the perception of a safety climate to the implementation of safety behavior is far from being exhaustive. Accordingly, this study aimed to explore the role of risk perception and safety knowledge in explaining the positive impact of safety climate before attending a training course (Time 0) and safety behavior after the training completion (Time 1). Data were collected at two time-points on a sample of *N* = 278 construction workers taking part in different safety training courses promoted by a vocational training organization in Northern Italy. The hypothesized relationships were tested using a serial mediation model bootstrapping approach. The obtained results indicated that the perception of a safety climate at Time 0 (T0) among construction workers is associated with higher risk perception and safety knowledge that, in turn, resulted in a higher implementation of safety behavior at Time 1 (T1). These findings contribute to the understanding of those factors that constitute a fertile ground for preventing injuries and accidents in the construction sector.

## 1. Introduction

Safety climate has been widely recognized as a primary predictor of workers’ safe behaviors [1,2]. Empirical evidence indicates that training initiatives could improve individual, group, and organizational effectiveness [3]. In the safety field, training could prevent and reduce the occurrence of injuries, illnesses, and accidents in the workplace. The extent to which the learning that stems from a training experience is transferred to one’s job (i.e., transfer of training) is significantly affected by the perception of a safety climate in the workplace [4]. The impact of the safety climate on objective and subjective measures of safety behavior has been observed across different occupational sectors and cultural contexts [5]. The purpose of the present research is to investigate the role of safety climate in stimulating the enactment of safe behaviors. More specifically, the current study aims to explore whether the enhancement of safety knowledge and a higher risk perception could explain the safety climate’s influence on the implementation of safety behavior among construction workers.

Linear relations between safety variables (safety climate, safety knowledge, risk perception, safety behavior) are well documented in academic research. This research was aimed to test these associations among workers from a different occupational sector (i.e., the construction sector) and a specific cultural context (i.e., the Italian socio-cultural context). Furthermore, the current study was aimed at testing sequential relationships between safety-related variables using a serial mediation model bootstrapping approach. The use of a systemic approach that simultaneously takes into account the role of personal and situational factors on acting safe behaviors adds value to this research. The purpose of gathering a deep understanding of the relationship between safety variables is valuable both from a theoretical and practical point of view. An enhanced awareness concerning the antecedents of safe behaviors is helpful in injuries prevention. Thus, the current study was aimed at offering suggestions for safety intervention management. The encouragement of safe practices among construction workers can rely on interventions at the organizational level (to improve safety culture) as well as at the individual level (to improve safety knowledge and risk perception). The present research tested the systemic effect of personal and environmental variables on safe behaviors. A joint intervention embracing these variables at different levels of analysis could reach a self-reinforcing reciprocal impact in boosting safe practices. Especially among organizations with limited financial resources, the adoption of a serial mediation model allows organizations to identify different targets for interventions aimed at fostering the antecedents of safe behaviors (i.e., safety climate, risk perception, safety knowledge).

### 1.1. From Safety Climate to Safety Behavior

Organizational climate is described as the combination of shared perceptions among employees concerning the procedures, practices, and behavior that are rewarded and supported in the workplace concerning a strategic focus [6]. When this strategic focus involves performing high-risk operations safely, the resultant shared perception is defined as a safety climate [7]. The concept of safety climate was introduced in 1980 to describe employee’s shared perceptions regarding the relative priority of safety in comparison to other organizational goals [8]. Organizational climate originates in individual members’ experiences and gradually turns into a socially shared perception ascribable to a group-level variable [9]. According to this definition, shared climate perceptions evolve as a result of ongoing member-leader and member-member interactions.

A safety climate entails different domains: safety policies, procedures, and practices, but also their relationship with productivity or efficiency [7]. The impact of climate perceptions could be explained by allowing employees to discern which behavior is expected, rewarded, and supported. If productivity is favored over safety and assumes a higher priority, employees are prompted to act consistently and attribute a secondary role to safety. From a practical point of view, a safety climate involves employees’ evaluation and their shared perception about the priority of safety compared to other competing priorities [10]. A safety climate entails a group-level variable that leads workers to share relevant features of their job and shape perceptions on the significance that management assigns to compliance with safety norms [11,12]. A large number of studies have assessed perceptions of a safety climate among construction workers and their associations with positive outcomes, to identify those mechanisms that could be buffered to reduce the injury rates [1,13,14]. Accordingly, prior results showed an inverse relationship between the perception of a safety climate and injury rates [15,16,17]. According to previous findings, safety training (considered as a component of the safety climate) could predict the adoption of safety behavior among manufacturing employees [11,18]. They also considered safety training as one dimension of the safety climate. Further conceptualizations of safety climate considered aspects such as safety supervision, management commitment, co-worker support, and safety training [19]. According to these scholars, safety supervision and management commitment had a positive impact on three different dimensions of safety behavior: safety initiates, personal protective equipment, and safety compliance.

A different definition comes from a noteworthy meta-analysis by Alruqi et al. [20]. It suggested five dimensions to assess the impact of safety climate on performance: management commitment to safety, supervisor safety role, safety rules and producers, training, and individual responsibility for health and safety. Previous studies were aimed at investigating the effect of safety culture on safe behavior considering the different facets of the construct. In contrast, the current research emphasized its general relevance in encouraging compliance with safety norms. In line with the results suggesting the positive effect of a widespread perception of a safety climate on the implementation of safety performance, a first aim of the current study was to test the predictive role of safety climate before attaining safety courses (Time 0) and safety behavior after the course completion (Time 1). Thus, the following hypothesis was formulated as follows:

**Hypothesis 1** **(H1).** *The perception of a safety climate at Time 0 (T0) will positively impact the implementation of safety behavior at Time 1 (T1) among construction workers.*

### 1.2. The Mediating Role of Risk Perception

In addition to a safety climate, the enactment of safety behavior also benefits from workers’ judgment concerning the frequency and severity of particular hazards occurring in the workplace (i.e., risk perception) [21]. People need to perceive the threat to respond to it appropriately. Pandit et al. [22] explored the impact of safety climate on hazard recognition and safety risk perception since both represent injury prevention’s critical factors. According to these authors, workers perceiving a more robust safety climate in their workplace also reported higher levels of hazard recognition and greater risk perception. In a similar vein, Bohm and Harris [23] emphasized that risk-taking behavior among construction site dumper drivers was influenced by situational factors, such as co-worker behavior or site safety rules, together with a poor safety culture that gave priority to production.

A large body of empirical evidence corroborated the assumption of a positive association between safety climate and a wide range of safety outcomes, including a higher perception of safety risks occurring in the workplace [2,16]. Overall, these findings highlight the need to delve deeper into the role of a safety climate in risk perception. Common examples of risk-taking behavior stemming from the underestimation of associated risks include the improper use of personal protective equipment, unauthorized use of construction equipment, and tampering with protective guards on construction tools [24,25].

Previous research showed the occurrence of a positive relationship between safety climate and behavior, suggesting a more significant risk perception, such as the use of personal protective equipment and compliance with safety rules and regulations, and their participation in safety knowledge sharing initiatives [26]. On the other hand, great familiarity and experience with work tasks can significantly reduce the hazard perception [27]. Thus, workers with considerable experience in their job roles usually feel incredibly confident when faced with dangerous tasks. In line with this evidence, empirical findings showed that workers perceiving their duties as potentially dangerous (i.e., risk perception) are prone to behave following safety rules and norms (i.e., safety behavior) [28]. For instance, Harbeck and Glend [29] revealed that young drivers characterized by a perception of higher risks in their daily activities tend not to engage in risky driving. In the field of public health and safety, Nie et al. [30] indicated that patients’ risk perception could be used to predict health-promoting self-care behaviors, such as diet, exercise, self-monitoring of blood glucose, adherence to treatment, foot care, and personal health responsibility. More specifically, risk perception also represents an essential factor in explaining safety behavior in the construction sector. Several studies investigated worker risk-taking behavior using a qualitative approach and recognized risk perception among construction workers as a primary predictor of their risk-taking behaviors [25]. In particular, risk perception among construction workers has a negative influence on their risk-taking behavior. For instance, these workers wear safety equipment and shoes when they believe this behavior could prevent severe injuries [31].

These findings could be particularly beneficial to the construction industry. Hence, safety management can provide safety training initiatives and media tools to enhance the awareness of the risk associated with specific work situations, such as falls and slips from heights among construction workers reporting a low level of risk perception [32]. This strategy could enhance their perception of harmful conditions and, in turn, reduce their proneness to display risk-taking behavior. As previously described, earlier studies investigated the influence of risk perception on young drivers and healthcare workers. The exploration of risk-taking behaviors among construction workers was limited to the employment of a qualitative approach. To date, quantitative research on the influence of risk perception on construction workers’ risk-taking behavior is far from being exhausted. Accordingly, the current study investigated the mediating role of risk perception in the relationship between safety climate at T0 and safety behaviors enacted at T1. Thus, the following hypothesis was tested:

**Hypothesis 2** **(H2).** *Higher risk perception is expected to mediate the impact of the T0 safety climate on T1 safety behavior.*

### 1.3. How Can Safety Knowledge Promote Safety Behavior?

Safety climate was studied in several hazardous work domains, such as chemical plants, chemical factories, manufacturing, and mining factories [33,34]. Griffin and Neal [35] found that manufacturing and mining workers’ perceptions of safety climate were meaningfully related to their safety knowledge and the extent to which they engaged in safe work behaviors. Hence, the perception of a safety climate could feed workers’ awareness about work-related risks and desirable skills, thus resulting in a greater implementation of safety behavior at work [36].

Burke et al. [37] hypothesized that safety knowledge, acquired primarily through training, is a crucial antecedent of different aspects of safety performance, such as using personal protective equipment and engaging in work practices to reduce risk. Similarly, Griffin and Neal [35] found empirical support to the positive relationships among workers’ level of safety knowledge and self- and supervisor-ratings of safety performance. Their model was based on noteworthy models of job performance [38,39], which differentiate between antecedents of performance, determinants of performance, and performance components. Neal et al. [40] considered safety climate as an antecedent of safety knowledge and safety motivation, which are defined as determinants of safety performance and safety compliance. Hence, the shared perception of an organizational climate particularly focused on safeguarding safety could result in a greater knowledge of this facet of individual work experience.

Accordingly, Vinodkumar and Bhasi [34] revealed that safety knowledge and motivation are crucial antecedents of the enactment of safety behavior. In their study in Indian chemical factories, these authors showed that safety knowledge and safety motivation explain the relationship between safety management practices and workers’ safety behavior. Therefore, safety knowledge and safety motivation were identified as drivers for more robust safety compliance and higher workers’ engagement in safety.

The impact of climate perception on knowledge could be multifaced. For instance, scholars argued that organizational climate could influence learning by increasing participation in training activities [41]. These findings suggest that safety climate should be classified as an antecedent of safety performance, and this association may be mediated by the enhancement of safety motivation and safety knowledge. These findings also supported the distinction between task and contextual performance within the safety domain [38]. Whereas task performance embraces those safety activities prescribed as part of the job, contextual performance refers to those safety activities aimed at supporting the broader organizational context. Previous research has mainly focused on concrete safety behaviors about task performance, such as wearing safety equipment properly.

In contrast, the current study tries to broaden the construct of safety performance to embrace activities such as participating in safety meetings and encouraging safety behavior among colleagues. Hence, it focuses on several contextual behaviors that enhance the compliance to safety rules by the entire work team, or the organization as a whole [35]. Previous studies also recognized a boosting role that organizational climate could play in fostering the relationship between safety knowledge and the enactment of safety performance [42]. In particular, the perception of a climate that stresses the transfer of safety training could strengthen the relationship between safety knowledge and safety performance. Overall, these empirical results concur in drawing understanding of safety-related issues as a strong predictor of safety performance and a product of shared perceptions of a safety climate. Previous investigations were conducted on chemical plants, chemical factories, manufacturing, and mining factories, especially in Extra-EU countries. In contrast, the current study adds to the knowledge of safety behavior among construction workers from a European context. According to the theoretical reasoning and empirical findings discussed, the following hypothesis was developed:

**Hypothesis 3** **(H3).** *The impact of the T0 safety climate on T1 safety behavior is mediated by a higher level of safety knowledge among workers.*

### 1.4. The Nomological Network of Safety Behavior

**Figure 1:**
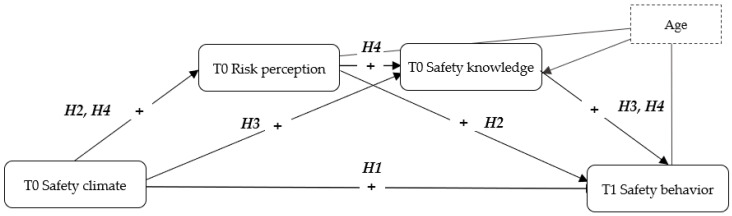
The hypothesized serial mediation model.

The evidence here discussed suggest that hazard recognition and risk perception among construction workers could be significantly improved through the attendance of suitable training interventions [43]. Supervisors with extensive work experience also had recurring opportunities to attend safety training interventions and, thus, reported a higher risk perception compared with students with shorter work experience and fewer training experiences. Hence, the ability to identify hazards in one’s work is meaningfully improved through work experience and fitting training initiatives. Thorough training is essential to prompt adequate levels of risk perception. As described in previous paragraphs, perceived risk is positively correlated to safety behavior [44,45]. The current study was focused on a sample of construction workers who attended training courses on safety and assumed that the perception of safety climate could foster the levels of hazard recognition and risk perception, in line with previous findings [22]. A higher risk perception, subsequently, is related to a higher awareness of rules and skills concerning safety issues. It is a significant predictor of the enactment of safety behavior and, in a complementary way, could discourage a risk-taking approach [25,31]. Consistent with this reasoning, this study is aimed to expand the current knowledge in this field of study. In particular, the hypothesized model embraces the relationship between safety climate, risk perception, and safety knowledge, also assessing their effect on safety behavior. The hypothesized serial mediation is depicted in Figure 1. As illustrated, the overall assumption of the current research was developed as follows:

**Hypothesis 4** **(H4).** *Safety climate at T0 is expected to trigger a higher occurrence of safety behavior at T1, through the subsequent mediation of higher risk perception and enhanced safety knowledge.*

## 2. Materials and Methods

The study was performed among workers taking part in courses organized by an institute for vocational training of the construction sector located in Northern Italy. In particular, research participants attended a 16-h course specifically designed for workers exposed to high safety risk. The course program included two theoretical lessons (4 h each) followed by two interactive classes based on practical activities and discussion conducted by the trainer. Before attending the course, participants were asked to fill in a paper-and-pencil questionnaire (T0), and the same survey was submitted at the end of the course (T1). At both the time points, the questionnaire included a cover letter reporting the aims and contents of the study. As defined by the Italian privacy law (Law Decree DL-196/2003) in terms of guidelines for personal data treatment, participants’ anonymity and information confidentiality were emphasized.

Moreover, informed consent was implied through survey completion. This research complied with the latest version of the Declaration of Helsinki [46]; thus, ethics approval was not compulsory, as per applicable institutional and national guidelines. Additionally, the current study did not involve any treatment, medical diagnostics, or procedures generating psychological or social discomfort among participants. A total of *N*= 278 workers completed the questionnaires at the two-time points. Most of them were men (88.8%) and ranged from 18 to 67 years old, with a mean age of 39.84 (*SD*= 10.63). Concerning their nationality, most participants were Italian (71.9%), 12% were Romanian, 2.9% were Moroccan. Overall, 88.1% of the sample consisted of workers from EU countries. The more prevalent employment contract was the permanent contract (65%), and most participants worked full-time (57.7%). Almost half of the sample was composed of workers with a college degree (45.4%), whereas 37.9% were primary education graduates, and the remaining 16.8% held a university degree. Moreover, the mean job tenure in the current company was 6.86 years (*SD* = 6.83).

### 2.1. Measures

As previously stated, participants filled in a self-report questionnaire before the beginning of the training course (T0) and immediately after the end of the course (T1). The implementation of a time-lagged design minimized the likelihood of common method variance and investigated the causal relationship among the study variables [47].

*Safety climate* before the course attendance (T0) was measured using the safety climate scale developed by Hahn and Murphy [12]. The scale consists of six items referring to workers’ perception of an organizational environment that emphasizes the relevance and encourages compliance to safety norms. A sample item is: “Workers and management work together to ensure the safest possible conditions.” Each item was rated on a five-point Likert scale ranging from 1 = *completely disagree* to 5 = *completely agree*. Cronbach’s alpha for the scale was α = 0.70.

*Risk perception* at T0 was measured using the scale built on the risk perception inventory [48,49] and consisted of six items assessing the perception of risks related to the construction industry-specific scenarios. A sample item is “In case of mistakes or dangers, I can manage the risk of accidents by myself.” All items were rated on a five-point Likert scale ranging from 1 = *totally disagree* to 5 = totally agree. The internal consistency of the scale yielded a Cronbach’s alpha coefficient of α = 0.65.

*Safety knowledge* at T0 was assessed with a self-constructed six-item scale developed in collaboration with course trainers. It aimed to evaluate one’s awareness of the hazards involved in the execution of activities specifically for the construction sector. A sample item is “Do you know why asbestos is harmful to health?” The response options varied on a five-point agreement scale from 1 = *completely disagree* to 5 = *completely agree*. In the present study, the reliability of this scale was α = 0.87.

*Safety behavior* after the training course (T1) was assessed with six items adapted from the safety compliance subscale developed by Vinodkumar and Bhasi [34]. A sample item is: “I use all necessary safety equipment to do my job.” Robson et al. [50] suggested that the current research conceived these behaviors as “immediate outcomes” promptly detectable after the training course. Participants were asked to indicate the extent to which they agree with each of the items, using a five-point scale ranging from 1 = *totally false* to 5 = *totally true*. Cronbach’s alpha for the scale was α = 0.63.

### 2.2. Strategy of Analysis

The research hypotheses were tested using a bootstrapping approach developed by Hayes [51]. This method is particularly suited with limited sample sizes because it computes a distribution using the observed data, from which statistical effects are estimated [52]. In the current research, model 6 from the SPSS macro PROCESS was applied to test a mediation model with multiple mediators operating in serial. This model evaluates all path coefficients at once, to derive the direct and indirect effects of the hypothesized model. Moreover, the bootstrapping method generated an estimate of the indirect impact, including a 95% confidence interval. When zero is not included in the confidence interval, one can conclude that the indirect effect of the independent variable (i.e., T0 safety climate) on the criterion variable (i.e., T1 safety behavior) is mediated by the proposed sequential mediators (i.e., T0 risk perception and safety knowledge). Based on previous empirical results, we decided to control for the potential confounding effect of participants’ age [53].

## 3. Results

### 3.1. Descriptive Statistics

Descriptive statistics for all study variables are reported in Table 1. All the associations among the constructs under investigation were significant in the expected direction, whereas participants’ age did not report any significant relationship. Furthermore, as indicated on this table’s diagonal, all the scales satisfied the criterion of 0.65 [54], except for safety behaviors, which report a slightly lower coefficient.

### 3.2. Model Testing

Table 2 reports the estimates of all the path coefficients and the 95% bias-corrected bootstrapped Confidence Intervals (95% CI) concerning the indirect relationships included in the hypothesized model. The perception of a safety climate at T0 (i.e., the independent variable) showed a positive association with risk perception (B = 0.21, SE = 0.05, *p* = 0.000, 95% CI [0.12; 0.31]) and safety knowledge (B = 0.17, SE = 0.08, *p* = 0.037, 95% CI [0.01; 0.33]). In addition, in the current sample a higher perception of a safety climate in the workplace at T0 had a positive impact on the implementation of a safety behavior at T1: B = 0.10, SE = 0.05, *p* = 0.044, 95% CI [0.01; 0.20]. This positive effect provided support to Hypothesis 1.

The obtained results indicated that workers’ risk perception at T0 was associated to a greater safety knowledge among workers (B = 0.32, SE = 0.10, *p* = 0.001, 95% CI [0.12; 0.52]) and reported a positive impact on safety behavior reported at T1 (B = 0.14, SE = 0.06, *p* = 0.029, 95% CI [0.01; 0.26]). In addition, T0 risk perception significantly mediated the effect of T0 safety climate on T1 safety behavior, with B = 0.03, SE = 0.01, 95% CI [0.01; 0.06]. This evidence supported Hypothesis 2.

Furthermore, T0 safety knowledge showed a positive relationship with T1 safety behavior, with B = 0.14, SE = 0.04, 95% CI [0.07; 0.21]. As assumed in the hypothesized model, T0 safety knowledge played a mediating role in the association between T0 safety climate and the enacted safety behavior at T1, with B = 0.03, SE = 0.02, 95% CI [0.01; 0.07]. This result provided empirical support for Hypothesis 3.

The overall model tested in the current research included T0 safety climate (i.e., the independent variable), workers’ risk perception (involved as the first mediator), safety knowledge (incorporated as the second mediator), and T1 safety behavior (i.e., the model outcome).

Interestingly, results indicated that risk perception and safety knowledge sequentially mediated the relationship between the perceived safety climate before attending the training activity (T0) and the implementation of safety behavior after the course completion (T1). The serial mediation model reported an indirect effect with B = 0.01, SE = 0.01, 95% CI [0.01; 0.03]. This evidence lent support to our last assumption, developed in Hypothesis 4. In particular, the T0 safety climate was associated with higher levels of risk perception, which led to stronger safety knowledge and, in turn, to a greater enactment of safety behavior at T1.

Workers’ age included as a covariate did not report any significant association with the constructs under investigation (Table 2).

## 4. Discussion

The present research was aimed to explore the role of safety climate in stimulating the enactment of safe behaviors. More specifically, the enhancement of safety knowledge and higher risk perception were hypothesized to explain the influence of safety climate on the enactment of a safety behavior among construction workers. The obtained results supported a positive impact of the safety climate at T0 on safety performance at T1. These findings concur with previous evidence suggesting a positive association between the widespread of an organizational climate on safeguarding the safety and consistent behaviors among workers and a negative relationship between the perceptions of a safety climate and injury rates in the workplace [14,15,16,17].

Furthermore, the obtained results contribute to the ongoing literature on those drivers that could be enhanced in order to promote safety conduct among workers. In particular, a higher perception of the workplace’s risks could stem from the spread of a safety climate in the organizational setting [22]. This enhanced awareness, in turn, is related to the comprehension of ways of behaving safely, which is reflected in the level of safety knowledge [34]. Overall, these findings concur with remarkable meta-analyses aimed to disentangle the relationship between safety climate and employee safety outcomes [2,55,56], which have highlighted solid relationships between safety knowledge, motivation, compliance, participation, and a lower occurrence of employee injuries. Furthermore, the current results are in line with previous evidence suggesting that safety knowledge explains the relationship between the perception of a safety climate, on the one hand, and the enacted safety behavior, on the other hand [1]. According to this evidence, two different facets of safety behavior are influenced by these antecedents: safety participation and safety compliance. Whereas safety compliance concerns adherence to explicitly stated safety rules and regulations, safety participation reflects safety-related organizational citizenship behaviors.

The current study has some limitations that should be acknowledged. First, the data collected at two points in time overcame the barriers of predominant cross-sectional designs in safety research. However, a study design, including four-wave data, would provide more robust empirical support to the serial mediation hypothesized. On the other hand, we followed the recommendation to test a half-longitudinal design to corroborate the occurrence of a causal relationship between the constructs under investigation [57]. A further limitation entails the internal reliability coefficient of the scale aimed to assess the implementation of safety behavior. This measure reported a Cronbach’s alpha coefficient slightly lower than the criterion of 0.65, which is traditionally considered as a heuristic [54]. On the other hand, this value could be considered acceptable due to the explorative nature of the current study.

Nonetheless, several scholars suggest that scales with item consistencies exceeding the minimum threshold of 0.60 can be used for study purposes in most social science research [58,59]. Moreover, the construct of climate is based on shared perceptions; thus, subjective measures are the most natural and suitable way to tap into this concept. On the other hand, we used a self-report scale to measure the implementation of behavior that is consistent with organizational safety rules and standards (i.e., safety behavior). An interesting venue for future research would be to replicate the current model using an objective measure of the safety behavior implemented by workers. This choice would allow reducing the likelihood of common method variance effects among the variables under investigation.

Furthermore, the sample under study was quite homogeneous and consisted of construction workers who took part in the training activities promoted by a specific institute for vocational training of the construction sector. This characteristic prevented generalizing the findings and conclusions of the current study concerning other occupational groups. Future studies could evaluate the postulated model’s occurrence among workers in further professional sectors of activity that generate the most fatal and non-fatal work accidents, such as the retail sector, the manufacturing sector, and the land and pipeline transport sector [60]. Accordingly, Elmoujaddidi and Bachir [45] noted that the perceived probability of accident occurrence and the potential severity of risk varies notably among workers according to their specialty. Fall from height risk is considered the most severe risk by bricklayers and blasters, but this risk was considered moderate by plumbers. The current research focused on risk perception with a specific focus on construction industry-specific scenarios but considering risk as a general construct. Future studies could test the same model using homogeneous groups of workers and focusing on particular risk perception (e.g., falling from the height, chemicals, or dust inhalation). As a further limitation, our sample is not balanced in terms of gender representativeness, since most participants were men. On the one hand, this feature is due to the occupational sector under investigation; on the other hand, a challenging venue for future research could entail the evaluation of gender as a potential moderator in risk attitude among workers [61].

### Practical Implications

The results of our study provided useful information to organizations interested in encouraging safety behaviors among employees. Organization leaders and supervisors play a vital role in influencing climate perceptions in the workplace by exposing workers to consistent policies and procedures that provide them with guidelines to focus their attention [62,63]. Therefore, a practical implication entails strategies aimed to enhance the perception of managers and supervisors that encourage attitudes and behaviors to prevent counterproductive work behavior, including unsafe practices [64].

Furthermore, the spread of safety climate and risk perception could benefit from organizations investing in interventions based on a participatory approach. These initiatives would agree with previous studies showing that participatory interventions aimed to improve safety rules can significantly impact risk perception [65]. On the one hand, all the empirical studies described as the background of the current research were based on traditional teaching methods. On the other hand, a large body of safety literature suggests that training with low engagement (by lectures, videos, or demonstrations) are typical in construction. However, they are minimally effective when compared with more engaging forms of instruction [66]. Additionally, training initiatives could be aimed at fostering workers’ ability to perform job crafting behaviors (e.g., seeking challenges), which can act as protective factors and that ease the implementation of safety behaviors [67].

Moreover, a deeper understanding of the relationship between safety climate, safety knowledge, and safety behavior could enhance the transfer of training. According to Smith-Crowe et al. [42], an organizational environment focused on the transfer of safety training would strengthen the relationship between safety knowledge and safety performance due to the alignment between workers knowledge and performance, on the one hand, and the goals of their organization, on the other hand. This suggestion would be consistent with Liu et al. [19], who considered safety training as a component of safety culture.

## 5. Conclusions

This study offers a deep understanding of the relations between safety variables in the Italian construction sector confirming and expanding previous research. From the practical point of view results show encouraging suggestions for individual and organizational intervention planning. The current study provides a more in-depth insight into the process that links the spread of a safety climate in the workplace and the enactment of a safety behavior among construction workers. Specifically, our study provided evidence suggesting that safety climate can positively influence risk perception and knowledge about safety, which in turn will result in safety behaviors enacted by construction workers. Overall, the current study contributes to the ongoing attempts to integrate the array of contextual and individual antecedents of safety behavior in the workplace.

## Figures and Tables

**Table 1:** Means, Standard deviation, Cronbach’s Alphas (in brackets), and Correlations among the study variables (*N* = 268).

	*M*	*SD*	*r*				
			1	2	3	4	5
**1. T0 Age**	39.84	10.63					
**2. T0 Safety climate**	3.89	0.68	−0.05	(0.70)			
**3. T0 Risk perception**	3.87	0.56	0.06	0.24 ***	(0.65)		
**4. T0 Safety knowledge**	3.50	0.91	0.01	0.18 **	0.23 ***	(0.87)	
**5. T1 Safety behavior**	3.65	0.57	−0.08	0.23 ***	0.17 **	0.29 ***	(0.63)

**Note.** ** *p* < 0.01; *** *p* < 0.001.

**Table 2:** Path coefficients and indirect effects for mediation models.

	Path Coefficients						Indirect Effects		
	To T0 Risk Perception (RP)		To T0 Safety Knowledge (SK)		To T1 Safety Behavior (SB)				
	b	SE	b	SE	b	SE			
Age	0.01	0.01	−0.01	0.01	−0.01	0.01			
T0 Safety climate (SC)	0.21 ***	0.04	0.17 *	0.08	0.10 *	0.05			
T0 Risk perception (RP)			0.32 **	0.10	0.14 **	0.06			
T0 Safety knowledge (SK)					0.14 **	0.03			
							**b**	**SE**	**95% CI**
Total							0.06	(0.02)	0.02; 0.11
SC → RP → SB							0.02	(0.01)	0.01; 0.06
SC → SK → SB							0.03	(0.02)	0.01; 0.07
SC → RP → SK → SB							0.01	(0.01)	0.01; 0.03

**Note.***N* = 278. * *p* < 0.05; ** *p* < 0.01; *** *p* < 0.001; 95% CI = 95% confidence interval using the bootstrap bias-corrected method using 5000 samples. Standard error in brackets.
